# The burden of chronic diseases across Europe: what policies and programs to address diabetes? A SWOT analysis

**DOI:** 10.1186/s12961-019-0523-1

**Published:** 2020-01-29

**Authors:** Angela Giusti, Marina Maggini, Sofia Colaceci

**Affiliations:** 1 National Centre for Diseases Prevention and Health Promotion, National Institute of Health (Istituto Superiore di Sanità), Viale Regina Elena 299, 00161 Rome, Italy; 2 National Centre for Drug Research and Evaluation, National Institute of Health (Istituto Superiore di Sanità), Viale Regina Elena 299, 00161 Rome, Italy; 3 Saint Camillus International University of Health and Medical Sciences, Via di Sant’Alessandro 8, 00131 Rome, Italy

**Keywords:** SWOT analysis, non-communicable diseases, policy, diabetes programmes, governance, healthcare, intersectoral collaboration, stakeholder participation

## Abstract

**Background:**

Promoting the well-being at all ages and reducing premature mortality from non-communicable diseases (NCDs) is a major target of the Sustainable Development Goals. In the frame of the JA-CHRODIS, a Strengths, Weaknesses, Opportunities and Threats (SWOT) analysis was conducted to provide different countries’ insights on what makes a policy/programme addressing NCDs applicable, sustainable and effective, with a focus on diabetes.

**Methods:**

A qualitative study has been performed using a SWOT analysis on policies/programmes at the national/federal or subnational level.

**Results:**

By March 2016, 14 SWOTs were conducted involving 11 European countries and 57 stakeholders and Ministries of Health, reporting and analysing a total of 44 policies. The main strengths, weaknesses, opportunities and threats have been outlined as well as and the main areas for governance improvement. A binding trans-sectoral approach is necessary to tackle the underlying risk factors of inequalities. The culture of disease prevention and health promotion is still low while the biomedical paradigm prevails. A systematic gender perspective is still missing. Sharing and exchange of best practices, as sponsored by the European Commission, is acting as a motivator.

**Conclusion:**

The SWOT analyses draw an overall picture of the complexity of designing and implementing good policies and programmes that are tailored to local needs. These results may apply to any context and can be used by decision-makers, managers, professionals and other stakeholders to focus on key issues, recognising areas for attention.

## Key points

- The paper analyses policies and programmes across Europe from the perspectives of local decision-makers and stakeholders
- The SWOT methodology is structured and allows a qualitative descriptive and cross-sectional analysis
- The results of this study may apply to different contexts in Europe and can be used by decision-makers, managers, professionals and other stakeholders when designing and implementing policies and programmes on non-communicable diseases, focusing on key issues and recognising areas for attention

## Introduction

Ensuring healthy lives and promoting well-being at all ages is a major target of the 2015 Sustainable Development Goals [1]. More specifically, target 3.4 aims “*to reduce by one-third premature mortality from Noncommunicable Diseases* [NCDs] *through prevention and treatment, and promote mental health and wellbeing*” by 2030. Already in 2011, the General Assembly of the United Nations, with support from the European Union, adopted a political declaration on the Prevention and control of NCDs. World leaders committed themselves to strengthening international cooperation, including collaborative partnerships in support of national, regional and global plans for the prevention and control of NCDs, through the exchange of best practices in the areas of health promotion, legislation, regulation and health systems strengthening, training of health personnel, and development of appropriate healthcare infrastructure.

The launch of the European Joint Action on Chronic Diseases and Promoting Healthy Ageing across the Life Cycle (JA-CHRODIS) in 2014 [2] and CHRODIS+ Joint Action on Implementing Good Practices for Chronic Diseases in 2017 [3] is a response to the objectives set by the United Nations [4] and the European Commission. The goal of these Joint Actions is to promote and facilitate a process of exchange, transfer and implementation of good practices among countries and regions, for effective action against chronic diseases.

In the frame of the JA-CHRODIS, a Strengths, Weaknesses, Opportunities and Threats (SWOT) analysis was conducted to give an overview of the current policies and programmes on diabetes, which was considered a case study on strengthening healthcare for people with chronic diseases. The aim was to provide the point of views and insights of different countries’ on what makes a policy/programme applicable, sustainable and effective from a public health perspective as well as from the stakeholders’ perspectives, what are the necessary preconditions for its implementation, and the lessons learnt from the experience.

## Methods

**Fig. 1:**
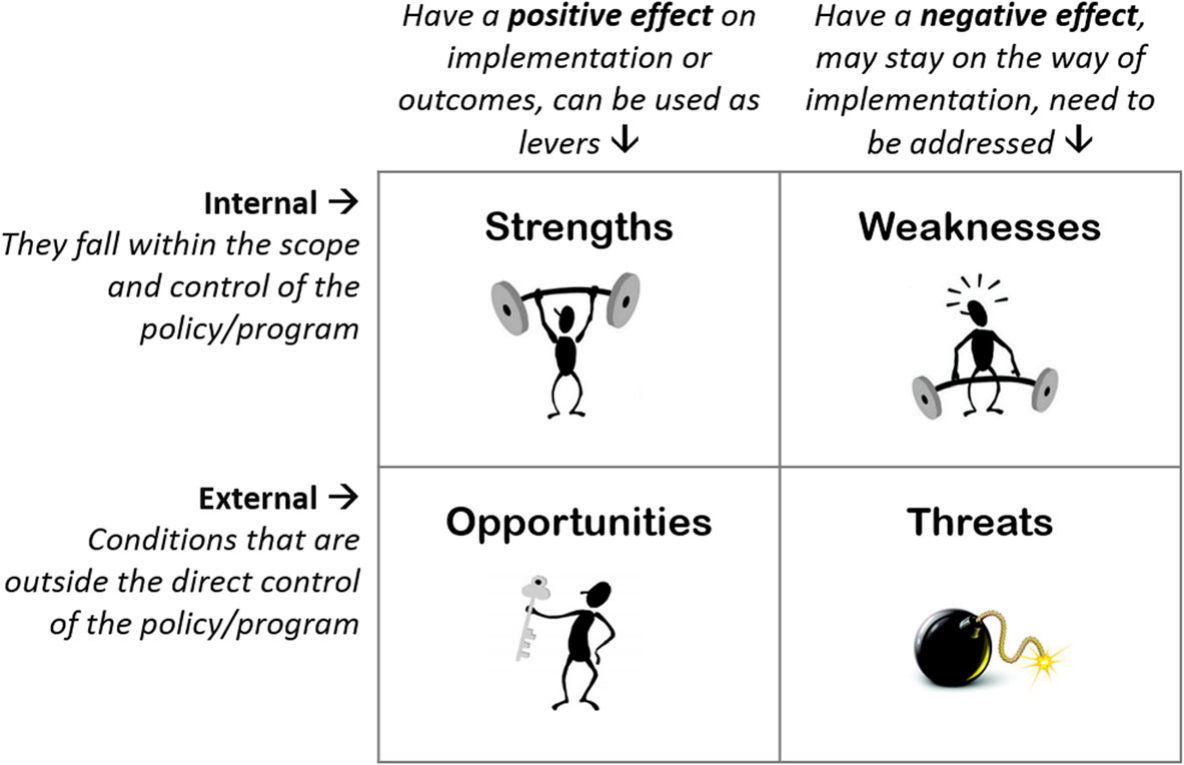
What is the SWOT analysis?

A qualitative study was carried out through a SWOT analysis, which is a strategic planning tool extensively used in business, community development programmes, health and education [5–7]. The SWOT aims to reveal positive forces that work together and potential problems that need to be recognised and possibly addressed. It enables participants to share their vision in a structured and intuitive way and to enrich the common perception. The SWOT methodology addresses and highlights all the characteristics, relationships and synergies among internal and external variables of a phenomenon (i.e. policy or programme) (Fig. 1). For this reason, the stakeholders involved in the analysis must have specific knowledge of the topic and an overview of the context. The analysis can be based on the single experts’ points of view or shared scenarios with other stakeholders, according to a participatory approach (i.e. Focus Groups, Workshop, Metaplan, World Café).

The SWOT methodology was presented, discussed and agreed during the third meeting of the WP7, held at the National Institute of Health in Rome. The partners were asked to include, in the analysis, five main current policies/programmes on prevention and care of diabetes as standalone policies/programmes or as part of a more comprehensive national plan (chronic diseases programme). The partners and participating experts were also asked to describe the successful strategies and the lessons learnt. Those partners who represent associations/organisations conducted the SWOT considering policies on specific topics. The level of analysis has been national/federal or subnational. If no policies were available in a country, the analysis addressed the external factors that could make the policy/programme feasible and sustainable or that might be considered as external threats.

In the JA-CHRODIS SWOT analysis, several dimensions could be explored, including planning, endorsement by policy-makers and stakeholders, implementation, organisational changes, partnerships, multi, inter or trans-sectorality, management, aspects relating to human resources, technology and information systems, coordination of care (i.e. multi or interdisciplinarity), funding, integration with other policies/programmes, support by laws or regulations, leadership, empowerment, capacity-building, monitoring and evaluation, and internal and external communication. According to the different phases of planning or implementation in the different countries, the SWOT could be ex ante, intermediate or ex post.

The phases of the data analysis were qualitative content analysis, deductive application of predefined categories and inductive development of new emerging categories. The analysis was conducted using NVivo 10.0 software for qualitative data analysis. All the texts of the SWOTs have been coded building up an interpretative model based on the categories described by the partners. The coding tree has thus been transformed and represented as a step-by-step model of the policy/programme development and implementation (Additional file 1: Figure S1). The ‘spiral model’ aims to describe the policy/programmes pathway, from context analysis through the implementation, ending in transferability and dissemination of good practices. The COREQ checklist [8] has been used for this report for the applicable items.

All authors declare no conflict of interest.

## Results

**Fig. 2:**
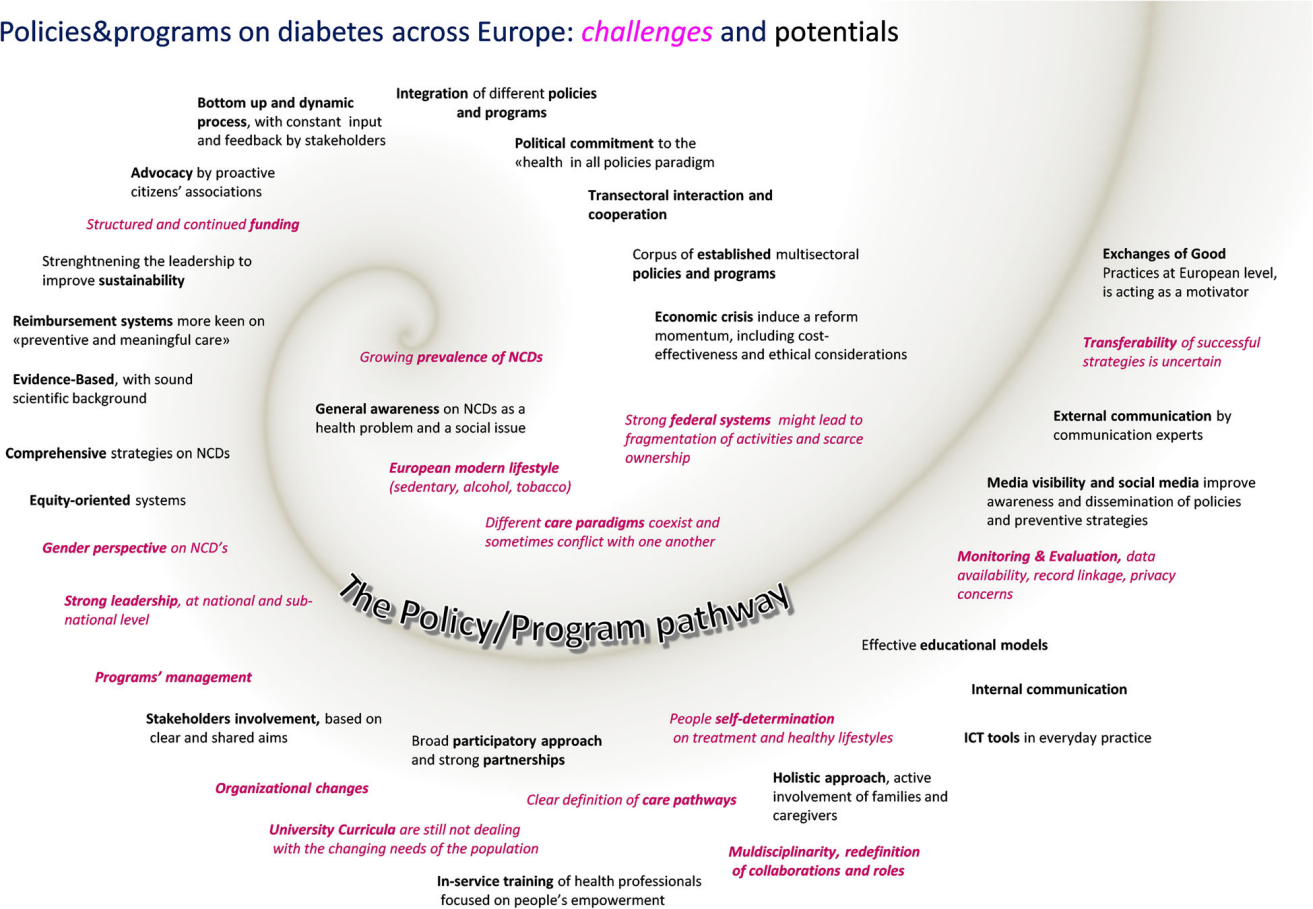
Countries involved in the SWOT analysis

By March 2016, 14 SWOTs were conducted and the data was analysed. A technical report was used to support skills-building seminars and is available on the JA-CHRODIS website [9]. SWOTs were conducted by 11 countries, namely Austria, Finland, France, Germany, Greece, Italy, Lithuania, Norway, Portugal, Slovenia and Spain. The European Wound Management Association, the European Institute of Women’s Health and the European Patients’ Forum/International Diabetes Federation Europe respectively analysed the management of the diabetic foot and education of professionals as a general overview across the EU; gender perspectives of national policies and programmes on the prevention and management of diabetes; and patients’ perspectives of national policies in Belgium. A total of 57 stakeholders and Ministries of Health in 12 countries contributed to the SWOT, reporting and analysing 44 policies. The participation methods were through email, face-to-face meetings, group video calls or individual calls; face-to-face meetings were used as a single methodology while the others were combined (Table 1, Fig. 2).

**Table 1:** Stakeholders involved and policies/programmes analysed

Number of stakeholders involved	57
Mean per SWOT	4.07 (1–10)
Number of policies/programmes included	44
Mean per SWOT	3.14 (0–6)
Methods of participation (*n* = 22, 14 SWOT)	
Email	10/22
Meeting	9/22
Group video call	2/22
Individual call	1/22

### Strengths and successful strategies

To be successful, a policy or programme needs to be built on a bottom-up and dynamic approach, being adapted regularly, with constant input and feedback by the stakeholders. The programmes should also be flexible enough to give a general framework for activities, which facilitates relatively free conduction of the project by different partners. As a result, new models and practices are developed bottom-up, based on local needs, resources and initiatives. Moreover, a national scale disease management programme can provide a general frame, while the subnational levels can develop their structured diabetes programmes, which consider regional differences, geographic distances in some less populated regions, and other specific characteristics of the local context. All national and local partners and stakeholders should be involved from the beginning of the planning and the partnership should be kept active throughout the process. Within the health sector, particularly important is the partnership of the medical associations and those of people/patients with chronic conditions. Health equity is often referred to in low socioeconomic and minority groups. The issue of gender should be considered on both national and European Union levels of policies and programmes. Partners reported a favourable reimbursement system of diabetes treatment and the universal accessibility of care as a successful strategy to address health equity. A strong scientific background was considered critical and it was determined that the guidance supporting the national and local programmes must be evidence-based, proactive, comprehensive and address the most common NCD risk factors, as most of the persons with chronic diseases suffer from multiple comorbidities. From the organisational point of view, a successful strategy includes the definition of the needed positions (e.g. diabetes nurses, podiatrists, psychologists, dieticians) and a strategic continuity of care. A clear description of the care pathways is needed, addressing specific groups and the areas of health promotion, diabetes prevention and treatment, including specialist and intra-hospital referral. In some cases, the care pathways are defined at the national level and supported by an information system at the national, subnational and local level. Remote consultation and shared medical electronic records facilitate access to the individual data by the person themselves and by the healthcare professionals. Regular monitoring and evaluation, with defined and shared outcomes and indicators, are important drivers for further programme implementation, using both quantitative and qualitative methods. Successful strategies include also population-level evaluation and a systematic media follow-up, including population awareness on diabetes and other chronic conditions. From the planning point of view, dividing the programme into subprogrammes has facilitated the efficient and coordinated conduct of the whole task. The definition of sound objectives on integrated care, shared among national and subnational levels, has been a leading starting point. Strong and efficient leadership is needed at the governmental, subnational and local levels, supported by information and communication technology tools for effective internal communication. Although structured and continued funding is difficult, different sources can be involved. In some cases, the municipalities and organisations invested their funds, engaging into the programmes. In this analysis, capacity-building is intended as the development and strengthening of human resources, focusing on people with diabetes and professionals. Good educational models and care strategies are essential for capacity-building and need to be shared with persons with diabetes and relevant associations.

### Weaknesses

Policies and programmes seem to be built mostly based on a medical paradigm and for single-disease treatment (i.e. type 2 diabetes only), whereas there is a growing need for a more holistic, cross-disease prevention and treatment approach. National NCD strategies need an action plan indicating the steps required for goals to be reached – this is not always provided. From a gender perspective, apart from pregnancy, there is not enough attention to how diabetes specifically affects women and no specific action is provided. The stakeholders’ involvement can be a challenge – where applied, coordination by patients’ organisations was not always welcomed by healthcare operators. In some cases, collaboration relied too much on a few motivated individuals, lacking long-term broader sustainability. As multiple sector involvement was not systematic, some important actors in society were not included (e.g. social and employment services). From an evidence-based practice perspective, the results from healthcare/translational research should be translated into practice more quickly and efficiently (e.g. delays in updating the guidelines). Several challenges related to the management of the programmes have been reported, including complex administrative rules on management procedures, poor coordination between local, regional and central authorities within the Ministry of Health, organisational, strategic and personnel changes, fragmentation and limited coverage of the existing diabetes prevention and care/cure programmes and clinical activities, difficulties to involve GPs into programme activities, allocation of human resources is sometimes inadequate, workload of the healthcare professionals, other healthcare professionals may fear that the programme implies a greater control over their work and may be resistant to change, and talented healthcare professionals get demotivated when they have to fight organisational obstacles or do not see results in real time. Budgetary constraints due to unexpected cuts in the health budget lead to severe cuts in well-established prevention programmes. Assessment, evaluation and comparison among different subnational levels are problematic. Educational programmes tend to be disease specific rather than addressed to persons with multimorbidity and lack of an integrated care approach (e.g. from hospital to home care), addressing diabetes management and lifestyle interventions. Some programmes are overwhelmingly hospital based, do not include a closest knowledge of the person, including family members and caregivers, and do not address the specific needs of the person after treatment.

### Opportunities

There is an increasing awareness across European institutions and healthcare systems that actions must be taken to address the prevention of chronic conditions and health promotion. Governmental support and general political commitment are indeed an opportunity, supporting the ‘health in all policies’ paradigm, NCD care, prevention and health promotion. Having a National Diabetes Plan is considered a key factor in the definition of a country’s health priorities. The economic crisis induces a health system reform momentum, where cost-effectiveness and ethical considerations are taken into account, moving towards a patient-centred, integrated and coordinated health and care approach. Society is becoming more sensitive to the prevention of diabetes as a social issue, reducing the social stigma, increasing the awareness of patients, and facilitating active participation in their care. Media visibility of the policies and programmes improves the awareness of professionals, patients, the general population and political decision-makers. The strategy gives an opportunity to explore, systematise and scale-up initiatives proven to be effective. Where organisational and clinical national guidelines are available, implementation, monitoring and modification to fit local context and endorsement by local professional societies is made easier. The development of information and communication technology tools in everyday clinical practice (e.g. e-records, e-prescription, e-protocols) will be a boost to the potential of monitoring and evaluation. Although still limited, prevention is now emphasised in the university training curricula, in the Continuous Education Programme, and new competence-based curricula are being developed for integrated chronic disease management and care. The existence of established policies or programmes within the health system and from different sectors (e.g. social sector, education) allows harmonised and target-oriented interventions. There is a huge unexploited potential in trans-sectoral interaction and cooperation between governments, local policy-makers, organisations, manufacturing and commercial industry to build infrastructures that promote healthy living.

### Threats

In countries with strong federal systems, one of the threats is fragmentation of activities, with scarce ownership and a definition of competences and responsibilities that is not always clear. This is critical in countries where primary care organisations are under the responsibility of municipalities, where fragmentation might be extreme. Policies themselves might be different in different areas of a country. For this reason, there is a variable uptake of guidelines, variable use of dedicated funds among levels of care and geographical regions, and national monitoring and evaluation may be rarely performed. To be effectively implemented, policies and programmes need a clear political commitment that might be challenged by political changes and the absence of long-term endorsement, particularly at the subnational level. At the local level, decision-makers do not always have knowledge or comprehension to make health policy decisions that have a multi-fold and long-term effect. The current economic crisis is challenging the public health systems across Europe, leading to large reforms and uncertainty about, for example, prevention and health promotion. The legislation on data security and privacy may hinder the assessment and evaluation process. As media attention and communication on NCDs are growing, it is sometimes claimed that people have the right to be and behave as they wish, and constant pressure from the authorities, e.g. to reduce obesity rates in the population, is a threat to a population’s autonomy. Moreover, mainly due to social media usage, laypeople are more active than ever before in discussions about lifestyle, questioning the authority of ‘experts’.

## Discussion

The study has collected data from countries that vary with regards to their geographical nature and administrative, political, social, cultural, environmental, economic and healthcare structure. Despite the differences, the emerging themes draw a scenario where the coexistence of old and new paradigms often conflicts, in a process that is exacerbated by the economic crisis. What makes a good policy or programme to address the population’s health and NCD has been described by WHO since Alma Ata declaration [10]. The Ottawa Chart [11] has further focused on the proactive and committed role that citizens and communities have on their health’s control. The results of the SWOT analysis show that a participatory Health in All Policies approach [12] supports implementation, assists in intersectoral cooperation, shared commitment and ownership, reduces mono-sectoral thinking and therefore leads to win–win solutions for complex problems. When the collaboration among different partners from different sectors becomes systematic, the networking may continue after the end of the project as an element of sustainability.

Indeed, real trans-sectoral involvement is challenging. When partners from different sectors participate in policy-making and programme planning, they often have different paradigms, interests, strategies, operational cultures and decision-making systems that may be highly consuming in terms of time, relationships and resources. However, a binding trans-sectoral approach is necessary to battle the underlying risk factors of inequalities. Despite improvements, the culture of disease prevention and health promotion remains low while the biomedical paradigm prevails. Where the emphasis in planning is on acute care and drug treatment, entrenching empowerment can be a huge task. As confirmed by other authors, a systematic gender perspective is still missing [13].

As a common ground, in European modern lifestyles there is widespread sedentarism and use of alcohol and tobacco [14]; the culture of eating and drinking is difficult to change, as it is to maintain healthy lifestyle changes. There is a need for specific laws protecting and promoting healthy lifestyles, also in consideration of the pressure of marketing strategies and interests of industry and economic lobbies, that may conflict with health outcomes and affect political decisions. Virtuous partnerships involving industries, civil society and media have been promoted by the European Commission to impact on NCD prevalence, e.g. by reducing the intake of salt, saturated fats, trans fats and added sugars, increasing the consumption of fruit and vegetables, reducing the impact of food marketing on children and breastfeeding, and reducing sedentary behaviour and inequalities [15].

There is a full range of initiatives addressing NCD prevention and health promotion that have been proven effectual and cost-effective. Despite NCD outcomes improving in Europe, “*there is scope for greater ambition*” [16]. Sharing and exchange of best practices, as sponsored by the European Commission through Programmes and Joint Actions, is acting as a motivator.

## Conclusions

The SWOT analyses have been developed across Europe, in countries that vary in political, administrative, social and health care organisations. The whole of all these considerations, thoughts, experiences and insights draws an overall picture of the complexity of designing and implementing good policies and programmes that are finely tuned to local needs. These results may apply to any context and can be used by decision-makers, managers, professionals and other stakeholders to focus on key issues, recognising areas for attention.

## Supplementary information

**Additional file 1: Figure S1.** Policies and programmes on diabetes across Europe: challenges and potentials.

## Data Availability

Not applicable.

## Abbreviations

JA-CHRODIS: Joint Action on Chronic Diseases and Promoting Healthy Ageing across the Life Cycle
NCDs: Non-communicable diseases
SWOT: Strengths, Weaknesses, Opportunities and Threats

## References

[1] United Nations Sustainable Development. https://www.un.org/sustainabledevelopment. Accessed 25 Feb 2019.

[2] Maggini M, Lombardo F, Caffari B, Giusti A, Icks A, Lindström J (2015). Diabetes: a case study on strengthening health care for people with chronic diseases. Preface. Ann Ist Super Sanita.

[3] Joint Action on Chronic Diseases, European Union. www.chrodis.eu. Accessed 25 Feb 2019.

[4] Political Declaration of the High-level Meeting on the Prevention and Control of Non-communicable Diseases. United Nations General Assembly. A/66/L.1. 2012.https://www.who.int/nmh/events/un_ncd_summit2011/political_declaration_en.pdf. Accessed 16 Jan 2020.

[5] Ghazinoory S, Abdi M, Azadegan-Mehr M (2011). SWOT methodology: a state-of-the-art review for the past, a framework for the future. J Bus Econ Manag.

[6] Pope C, Mays N (1995). Qualitative research: reaching the parts other methods cannot reach: an introduction to qualitative methods in health and health services research. BMJ.

[7] Pope C, Ziebland S, Mays N (2000). Qualitative research in health care. Analysing qualitative data. BMJ.

[8] Tong A, Sainsbury P, Craig J (2007). Consolidated criteria for reporting qualitative research (COREQ): a 32-item checklist for interviews and focus groups. Int J Qual Health Care.

[9] Diabetes: A Case Study on Strengthening Health Care for People with Chronic Diseases. SWOT Analysis. Technical report, Joint Action on Chronic Disease (JA-CHRODIS); 2016. http://chrodis.eu/wp-content/uploads/2017/01/guide-for-national-diabetes-plans_final.pdf. Accessed 16 Jan 2020.

[10] World Health Organization. Declaration of Alma-Ata. International Conference on Primary Health Care. Alma-Ata; 1978. https://www.who.int/social_determinants/tools/multimedia/alma_ata/en/. Accessed 16 Jan 2020.

[11] World Health Organization. The Ottawa Charter for Health Promotion. In: International Conference on Health Promotion. Ottawa; 1986. https://www.who.int/healthpromotion/conferences/previous/ottawa/en/. Accessed 16 Jan 2020.

[12] Leppo K, Ollila E, Peña S, Wismar M, Cook S (2013). Health in All Policies. Seizing Opportunities, Implementing Policies.

[13] Frøen JF, Staines A, Vrijheid M, Casas M, Delnord M, Fiberg IK, van Gent D, McQuinn S, Zeitlin J (2016). Invisibility: the health information gaps affecting women, children and adolescents in Europe. Eur J Pub Health.

[14] Eurostat, Health Determinants - Lifestyles - Statistics Explained. https://ec.europa.eu/eurostat/statistics-explained/index.php?title=Category:Health_determinants_-_lifestyles. Accessed 25 Feb 2019.

[15] Jakab M, Farrington J, Borgermans L, Mantingh F (2018). Health Systems Respond to NCDs: Time for Ambition.

[16] EU Platform for Action on Diet, Physical Activity and Health. Public Health, European Union. https://ec.europa.eu/health/nutrition_physical_activity/platform_en. Accessed 25 Feb 2019.

