# Correction to: The burden of chronic diseases across Europe: what policies and programs to address diabetes? A SWOT analysis

**DOI:** 10.1186/s12961-020-0541-z

**Published:** 2020-03-12

**Authors:** Angela Giusti, Marina Maggini, Sofia Colaceci

**Affiliations:** 1 National Centre for Diseases Prevention and Health Promotion, National Institute of Health (Istituto Superiore di Sanità), Viale Regina Elena 299, 00161 Rome, Italy; 2 National Centre for Drug Research and Evaluation, National Institute of Health (Istituto Superiore di Sanità), Viale Regina Elena 299, 00161 Rome, Italy; 3 Saint Camillus International University of Health and Medical Sciences, Via di Sant’Alessandro 8, 00131 Rome, Italy

**Correction to: Health Res Policy Sys**

**https://doi.org/10.1186/s12961-019-0523-1**

It was highlighted that in the original article [1] Fig. 2 was incorrect and the link for the Additional file 1 was missing. This Correction article provides the correct Fig. 2 and the Additional file 1 with its link. The original file has been updated.

**Fig. 2:**
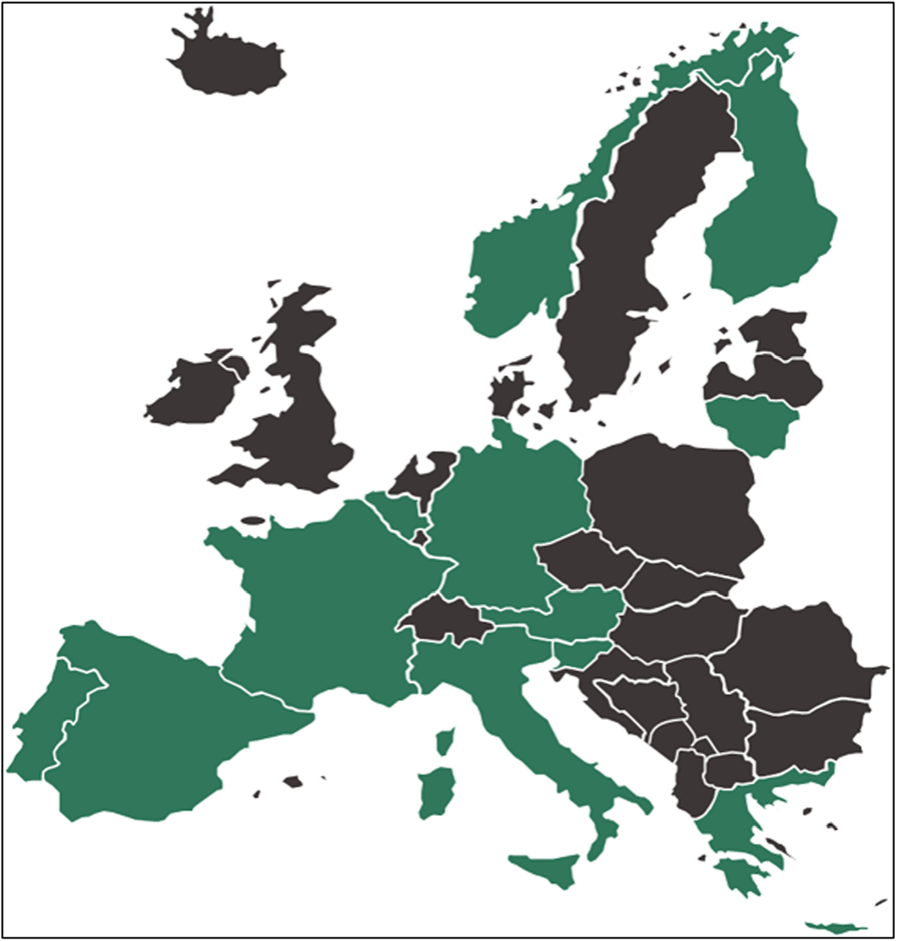
Countries involved in the SWOT analysis

## Supplementary information

**Additional file 1:Figure S1.** Policies & programs on diabetes across Europe: challenges and potentials.
