# Supplementary figures and images for: Correction to: The burden of chronic diseases across Europe: what policies and programs to address diabetes? A SWOT analysis
Source: Health Res Policy Syst. 2020 Mar 12;18:31. doi: 10.1186/s12961-020-0541-z (PMC7066759; doi:10.1186/s12961-020-0541-z)

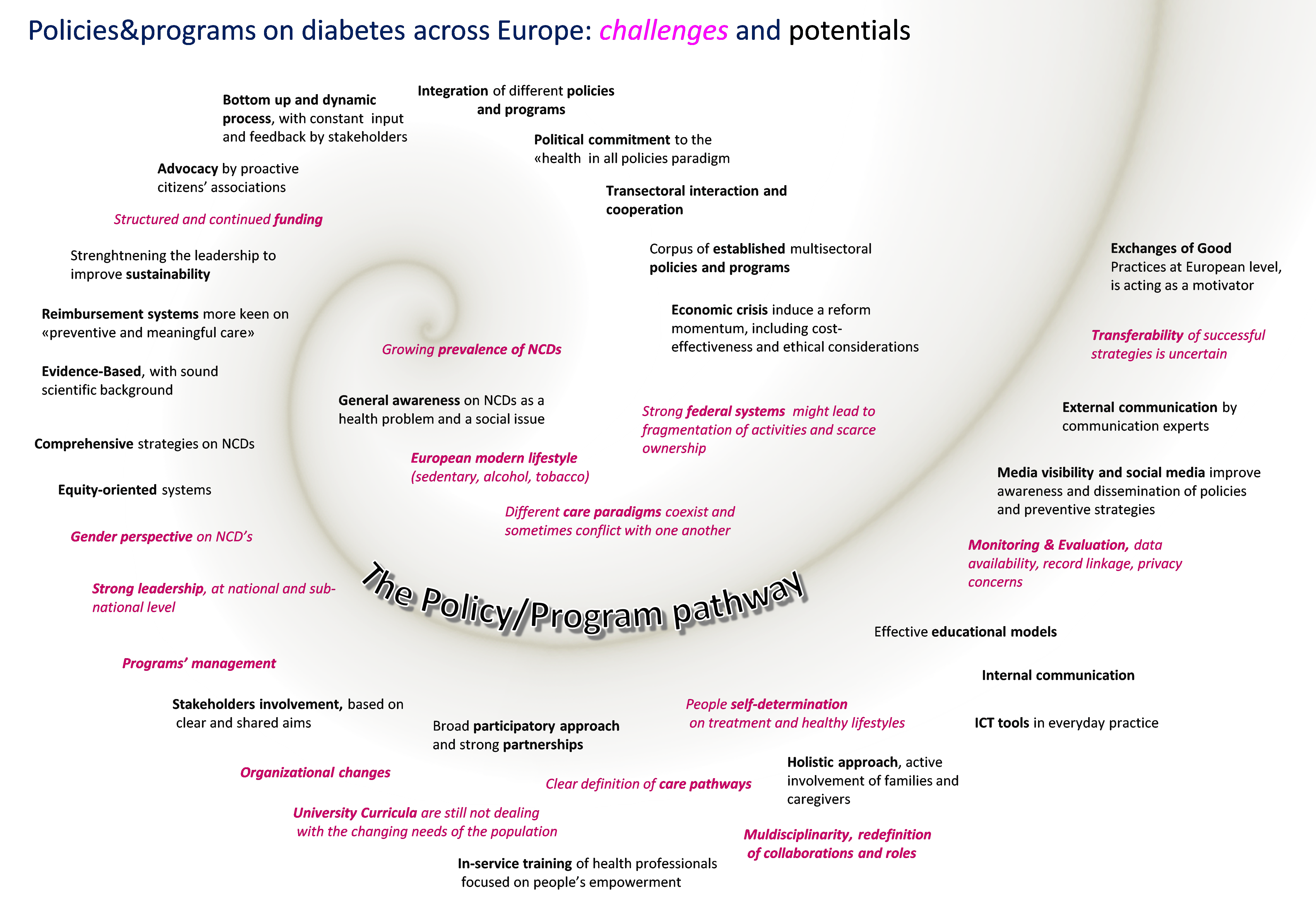

Supplement: Supplementary file 1 — Additional file 1:Figure S1. Policies & programs on diabetes across Europe: challenges and potentials. [file 12961_2020_541_MOESM1_ESM.tif]
